# Stitches in the Heart: A Small History of Scars

**DOI:** 10.1080/1472586X.2021.1915177

**Published:** 2021-06-08

**Authors:** Christina Lammer

*In my visual essay, the limits of representation shall be tested by means of multiple exposure filmmaking. Drawing on the arts-based research project Visceral Operations/Assemblage (2019–23), I use my 16 mm Bolex camera to penetrate the hardly tangible occurrences in the first week of November 2020: the terror incident in the heart of Austria’s capital city Vienna and the second lockdown during the COVID-19 pandemic. For this I will stitch together atmospheres in the surgical operating theatre, in the urban environment and at home. Vulnerability is a central issue that shall be addressed aesthetically. Thus, I will elaborate a small history of scars.*

## HEARTPLAY

A: May I lay my heart at your feet.

B: If you don’t make a mess on my floor.

A: My heart is clean.

B: We’ll see, won’t we.

A: I can’t get it out.

B: Would you like me to help you.

A: If you wouldn’t mind.

B: It’ll be a pleasure. I can’t get it out either.

A *cries*.

B: I will remove it surgically. What have I got this penknife for anyway. We’ll have this sorted out in no time. Work will keep you from despair. Right, there we are. But this is a brick. Your heart is a red brick.

A: Yes, but it beats only for you.(1981)

A *beats* B *to death with the brick*.

(Addition, July 1991)

(Heiner Müller)

The human heart consists of knotted tubes through which the living flows are propelled onwards. It beats silently in the dark chamber of the chest. Oxygenised blood, coming from the lungs, is transported through arteries and veins mostly unperceived. A mode of disappearance of our inner rhythmic alternation is at work, a central absence of the visceral processes. Inspired by philosopher Drew Leder’s study *The Absent Body* (1990), ‘I receive the surrounding world through my eyes, my ears, my hands. The structure of my perceptual organs shapes that which I apprehend’ (1). I will introduce a concept in which the heart as carnal material of modification and as delicate fabric from which love is made are inextricably intertwined with each other.

The circulation of energy in the body’s insides arises from a physical necessity. Through the respiratory system air is brought from the environment to the lungs. As we inhale oxygen enters the body and diffuses into the blood. Carbon dioxide waste is expelled as we breathe out. A continuous exchange of gases takes place. According to Stephen Porges, originator of *The Polyvagal Theory* (2017), ‘The vagus [nerve] facilitates the diffusion of oxygen into the blood by rhythmically modulating the blood flow and the resistance of the bronchi’ (140). Porges outlines ‘an integrated physiology that is not only regulating health, growth, and restoration but also fostering and supporting social interaction to create safety for the individual’ (141). Feeling safe is the critical feature of this approach.

The first language we learn is recognising and classifying touch. Physical contact conveys safety, social engagement and closeness. The COVID-19 pandemic, particularly the rule that we must not touch, hence, has threatening implications. Sociability is vital for life. After birth touch regulates breathing and the temperature balance in the body. When babies are not touched they do not grow and neither do they gain weight. The physiology of the touch stimulus is all-embracing.

Thus, I like to begin with a few manual techniques which inform my way of multiple exposure filmmaking: stroking, kneading, rubbing, rolling, pressing, shaking, stretching and vibrating. I am a trained massage therapist. This craft requires the skill to feel into the body as I have outlined in *The Camera is the Massage*, the artist pages of the *Crisis* issue of the *Millennium Film Journal* (Lammer 2020, 114–21). Giving a massage supports the receiver’s natural ability to heal. In my personal therapeutic style, I combine traditional Asian, classical Western forms of bodywork and breathing. Using the client’s cycle of respiration is an essential part of any treatment. Feminist philosopher Luce Irigaray writes in *The Forgetting of Air in Martin Heidegger* (1999), ‘to breathe […] means to be’ (62). Ex- and inhaling are modes of exchange with the environment:

Entrusting to the other the very rhythm of their breathing. Welcoming the loss of the measure of their breathing so as to discover for it a new range. Expiring in the other so as to thence be reborn more inspired. Putting language, the precinct of Being, into danger so that it might regain its voice. Its song. (177)

Social contact, breathing and heart rate variability are interconnected. Our feelings, says Porges, are dependent on our physiological state and on the autonomic nervous system. Compassion, for example, requires turning off defences. ‘Our nervous system evolved to detect certain features in the environment’ (Porges 2017, 147). *Neuroception*, according to the neurophysiologist, is the body’s ability to discover risk outside of the realm of awareness. Physiology colours our perception of the world and enables people to be interactive.

People need analogue face-to-face encounters without fear. Due to the coronavirus, however, the body has become a dangerous terrain. The daily news of the pandemic, dependent on the medium of transmission, generate anxieties. For the first time in evolution we are forced to keep a distance from each other. Any form of touching is to be avoided. The absolute absence of any bodily encounters leads to a physical state of high alarm. In the Western world, regardless of SARS-CoV-2, seeing clearly dominates in the hierarchy of the senses. The sense of sight allows to keep a distance. Whereas touch and sound work well with proximity.

The interrelatedness of looking, touching and vocalising is of particular interest in my sensory ethnographic research at the clinic. In his study *The Life of the Senses* (2015), anthropologist Francois Laplantine associates the notion of multiplicity with processes of folding. Folds indicate a change in the relatedness of surface to depth. Different layers are conjoined through their closeness to each other: hand to tissue, tissue to hand, in a contiguous coupling. The flesh of the hands and the texture of the skin are intimately related. Increasing elasticity and flexibility are among the positive effects provided by these targeted operations performed by hand. My process-oriented work with 16 mm multiple exposure colour and black and white film is an attempt to explore the sensitivity of the photographic membrane. For this I compare the human body with the filmstrip. Light penetrates into the skin of film.

Compassion – the connective tissue between clinical personnel and patients – was the subject of my explorations in a variety of cinematic research projects, which I have realised at the *General Hospital* in Vienna over the last two decades. I consider *Einfühlung*, the German term for *empathy*, as a malleable choreographic model that emerges in communion.

Cameras expand my capacities to register the gestures through which we can feel our ways into existing human and more than human conditions. At the beginning of my fieldwork at the clinic, I used simple mini-DV consumer camcorders. Currently I operate with semi-professional digital video and 16 mm mechanical vintage cameras.

The sense of touch plays an important role in my research and art work. For me cinema is a multisensory medium which allows me to interact with complex environments. The camera equally extends the hand and the eye, quite comparable with the brush of a painter or even with the knife of a surgeon. The skin of analogue film, its light sensitive emulsion, can be moulded like clay. While the membrane is mechanically guided through the apparatus, a mutual interaction takes place, a multilayered metabolic processing. Through the aperture of the lens – the mouth of the cinematic device – a certain amount of light is conducted. Just as much as the filmic mucosa is able to digest.

*Making Contact* (2004)^1^ is a short video in which the interconnected nature of bodies, materials, sterile garments, imaging technologies and instruments is represented as an essential part of the operations in interventional radiology where minimally invasive and catheter surgeries are performed (Lammer 2007, 91–116). For this thin probing wires are navigated through the tiniest blood vessels. Realtime fluoroscopy images help the surgeons to find their ways inside of the body’s finest tubes and structures. They even reach into the most delicate capillaries in the very periphery of the vascular system.

RADIOLOGIST: Please don’t move, don’t breathe. Don’t move, don’t breathe. Don’t move, don’t breathe.

ASSISTANT: Is everything okay with you?

PATIENT: Yes, though I don’t get enough air.

RADIOLOGIST: Why? Is breathing painful for you?

PATIENT: No, you did not say that I may breathe again.

*The team bursts into laughter*.

RADIOLOGIST: If I do not say anything, please only stop breathing for ten or 15 seconds or until the noise of the machine is away.

The attention of the operator roams from a digitally rendered visible roadmap of veins and arteries on a monitor to the person under treatment. Verbal communication is neglected during the operation.

The radiologist’s attentiveness is fully absorbed by the virtual landscape of the patient’s circulatory system. According to Leder, ‘There is a corporeal intertwining at the heart of such a craft’ (166). Whereas the patient’s visceral flows are set into scene by means of digital technologies, the physician’s eyes rest on the screen in front of him while his hands sensitively manoeuvre material components in and out of the most tangled inner rivers.

Hygiene precautions which are usually applied in the surgical theatre are currently intertwined with the modes of how we personally engage with each other. Touch and droplet transferrals of pathogens easily occur through direct contact with contagious people. As a consequence, social life needs to be brought to a standstill in order to avoid the spread of the coronavirus. Breathing changes in such a hostile situation. All living beings are impacted, though the animal and vegetal world much less so. We stay at home–in isolation–and the earth exhales.

A masked fight takes place, against an unknown invisible but nevertheless merciless intruder that has the potential to kill us. Tim Ingold writes in *Correspondences* (2021), ‘A world in which every communication is over almost before it begins, reducing life to a succession of instants, is simply not sustainable’ (4). Wearing a mouth-nose protection is unsociable. Our basic modes of expression are only possible to a very limited extent. ‘No living being, however, can persist indefinitely, nor can it carry on its life in isolation’ (10). This reminds me on what Luce Irigaray has outlined, in dialogue with Michael Marder, in *Through Vegetal Being* (2016):

Breathing is the first and the last gesture with regard to life, but to recover one’s breath in Paris, the city in which I was living, was not an easy task. I left the city for the woods or the mountains as often as I could, in order not to stay in a house or a studio but to walk outside in open air. […] The vegetal world was my favourite dwelling again, but, at least in the beginning, more as a place essential to my survival than as the Garden of Eden. (21)

**FIGURES 1–3.**
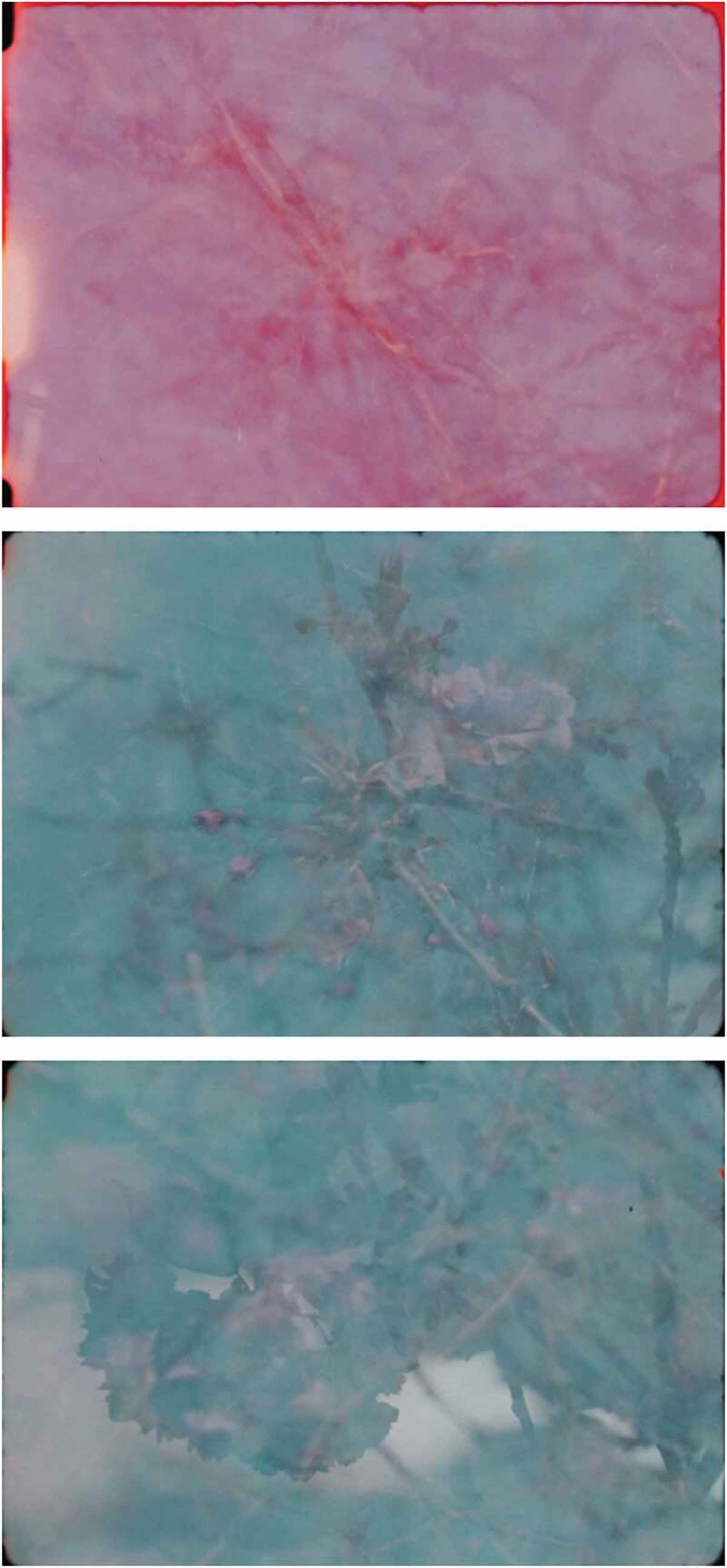
Christina Lammer, *Blooming Cherry Tree* (2020), still images of 16 mm multiple exposure short film, Vienna, Austria

I live in the centre of Vienna, Austria. Most of the parks and gardens in town were closed during the first lockdown in March and April 2020. Thus, I spent a part of the day in the courtyard which hosts a wonderful *Cherry Tree* (2020)^2^ that was just about to bloom in all its glory (see Figures 1-3). The blossoming of the tree gave me comfort and hope. I took my 16 mm camera and carefully accompanied its slow transformation for 24 days. The procedures turned into an enjoyable daily ritual that was performed outside in the fresh air.

A man in a white working overall paints puffy aerial structures, clouds and dark blue whirlwinds, drawn together by delicate threads, on a large-scale paper canvas. He works in an artist studio, far away from his usual area of operations, the *Cardiac Surgery Department* at the *General Hospital* in Vienna.^3^ Wilfried Wisser is cardiac surgeon. He, art historian, curator and physical theatre artist Tamar Tembeck, and I performed movement and painting workshops together at *artist-run centre OBORO* in Montreal, Canada. The mysterious landscapes painted by the physician represent his intraoperative perception of a heart valve. Associations with air and water, the essential elements life is made of, are quite intended.

**FIGURES 4–6.**
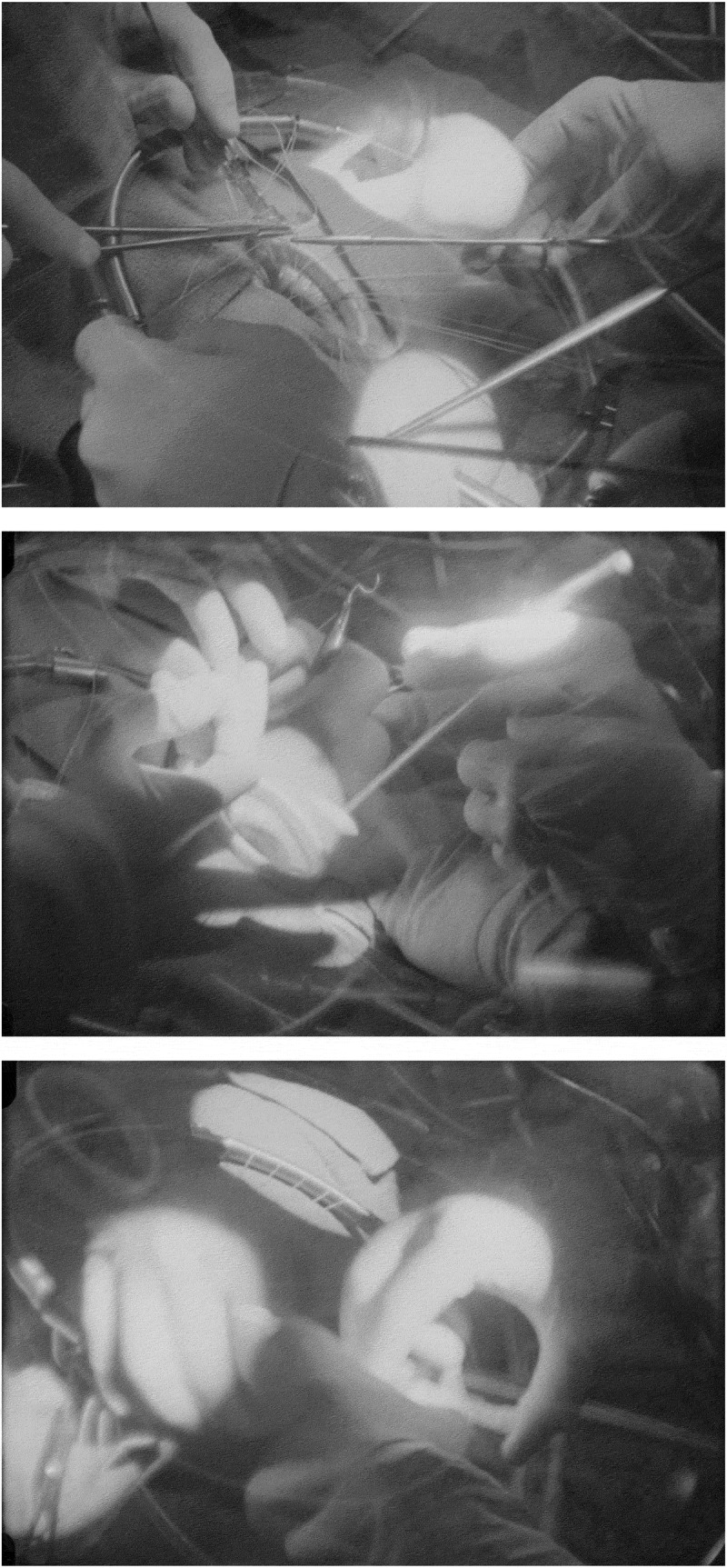
Christina Lammer, *Stitches in the Heart* (2019), still images of a 16 mm multiple exposure short film, Vienna, Austria

Pulsating light illuminates the space. A film projector is being fed. Hands dance rhythmically on the canvas. I trace the performance at home in my living room that has been forcibly converted into a workshop during the pandemic, far from the operating theatre where I filmed an intervention on the heart a few months earlier. In *Stitches in the Heart* (2019),^4^ a black and white multiple exposure film of a minimally invasive cardiac surgery, I focus on processes of sewing and hand gestures (see Figures 4-6). An orchestration of hands, bodies and material components unfolds. With the invention of new medical imaging technologies, the surgical craft is patterned in novel ways. Through the digital extension of the operator’s body by means of endoscopes and three-dimensional videography skills like making knots are most challenging. Basic procedures need to be relearned. Knotting is about forces and materials, sensory perception, movement and gesture.

Visceral interstices are touched and manipulated, joining together a scientific understanding of materials and a knowledge of flesh, of delicate layers growing out of experience. I explore the tying of knots as theatre of the hands. Circular manoeuvres are performed in a choreography of looping, operations of touching and making contact. The living body, according to Francois Laplantine, ‘grows, loses vigour, but also falls ill, shrinks, withers, bends, becomes stooped, and reddens when it becomes angry or is ashamed, trembles and grows pale when it is afraid’ (103). The viscera usually remain absent from perception and, up to a certain extent, strange within us.

**FIGURES 7–9.**
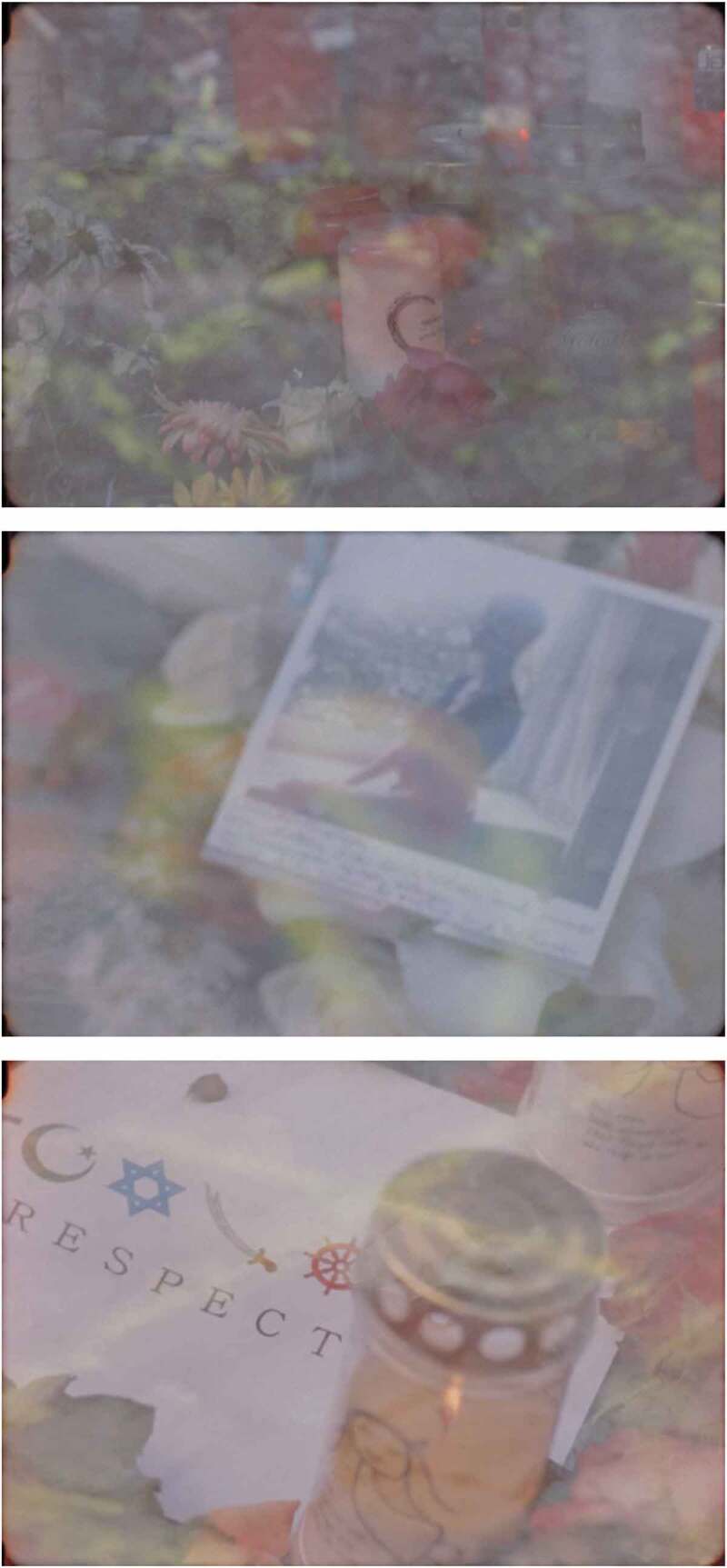
Christina Lammer, *All Souls* (2020), still images of a 16 mm multiple exposure short film, Vienna, Austria

Driven by an unbearable numbness that tended to seize me when I watched the horrible news of an ongoing terrorist attack in the centre of Vienna on television on the night of the *All Souls* (2020)^5^ day, I felt the urge to do something (see Figures 7-9). The densification of threatening events which rolled over us in just a few hours like shockwaves: a just proceeding act of terrorism in my home town, the beginning of the second lockdown and, last but not least, reports about the already ongoing US presidential election, left me breathless.

Nevertheless, I took my 16 mm vintage camera for a walk a few days later. I found Vienna’s heart at the first district deeply wounded. Bullet holes in windows and walls, traces of blood on the ground, lit candles and flowers in memory of those killed.

The image of the heart as red brick, entirely deprived of sensibility, haunts me. *Heartplay* can be perceived in multiple ways. The textures of loving, living and dying are examined with a sharp blade by theatre director and writer Heiner Müller. Drawing on his poetic probes, I aim at further elaborating manual techniques of feeling into the other. Love is homesickness, Freud says in his essay on the uncanny. Meditating about the murderous terror attack that occurred a few days earlier, I linger at the scene and pause for a while. My hands are hidden in the depth of a changing bag. I rewind 30,5 metres of 16 mm emulsion film in absolute darkness. In silent commemoration of the victims.

## NOTES

[1] See Christina Lammer, *Making Contact* (2004), Mini-DV, experimental video, 20 minutes. For a copy of the video see https://vimeo.com/483483329.

[2] See Christina Lammer, *Blooming Cherry Tree* (2020), 16 mm, colour, four minutes, 18 f/s, multiple exposure (24 exposures). For a scan of the film see https://vimeo.com/413495393, password: corporealities.

[3] See Christina Lammer, *Wrapping Around the Vulnerable* (2021), video essay, 20 minutes. For a copy of the video see https://vimeo.com/518621071, password: corporealities.

[4] See Christina Lammer, *Stitches in the Heart* (2019), 16 mm, black and white, four minutes, 18 f/s, multiple exposure (three exposures). For a scan of the film see https://vimeo.com/381168259, password: corporealities.

[5] See Christina Lammer, *All Souls* (2020), 16 mm, colour, four minutes, 18 f/s, multiple exposure (four exposures). For a scan of the film see https://vimeo.com/481691733.
